# Moderating effect of a sodium-rich diet on the association between long-term exposure to fine particulate matter and blood lipids in children and adolescents

**DOI:** 10.1186/s12887-024-04896-8

**Published:** 2024-07-20

**Authors:** YuHeng Cao, YunJie Liu, MengTing Ma, JiaRui Cai, MengMeng Liu, Rui Zhang, YunDi Jiang, Ling Yan, YueRan Cao, ZhenMi Liu, JiaQiang Liao

**Affiliations:** 1 Department of Maternal and Child Health, West China School of Public Health and West China Fourth Hospital, Sichuan University, Chengdu, 610041 Sichuan China; 2 Department of Epidemiology and Health Statistics, West China School of Public Health and West China Fourth Hospital, Sichuan University, Chengdu, 610041 Sichuan China; 3 Sichuan Center for Disease Control and Prevention, Nutrition and Food Hygiene Institute, Chengdu, 610041 Sichuan China; 4 School of Public Health, Faculty of Medicine, Imperial College London, SW7 2BX London, United Kingdom

**Keywords:** PM_2.5_, Blood lipids, Sodium-rich diet, Urinary sodium, Children, Adolescents

## Abstract

**Background:**

Several studies reported that exposure to higher levels of fine particulate matter (PM_2.5_) was associated with deteriorated lipid profiles in children and adolescents. However, whether a sodium-rich diet could modify the associations remains unknown. We aimed to examine the associations of long-term exposure to PM_2.5_ with blood lipids in children and adolescents, and further examine the effect modification by dietary and urinary sodium levels based on a multi-community population in China.

**Methods:**

The 3711 study participants were from a cross-sectional study, which interviewed children and adolescents aged 6 to 17 years across Sichuan Province, China between 2015 and 2017. Blood lipid outcomes including blood total cholesterol (TC), high-density lipoprotein cholesterol (HDL-C), low-density lipoprotein cholesterol (LDL-C), and triglycerides (TG) were assessed. Information on daily dietary sodium consumption was estimated with a semi-quantitative food frequency questionnaire (FFQ), and urinary sodium was used as an internal exposure biomarker. A linear regression model was applied to estimate the associations of prior 2-years’ average exposure to ambient PM_2.5_ with blood lipids. The effect modification by dietary and urinary sodium was examined by stratified analyses.

**Results:**

The participants from rural areas had higher levels of daily sodium consumptions. The results of multivariable regression analysis indicated that per 10 μg/m^3^ incremental change in PM_2.5_ was associated with a 1.56% (95% confidence interval 0.90%—2.23%) and a 2.26% (1.15%—3.38%) higher blood TC and LDL-C levels, respectively. Among the study participants with higher levels of dietary sodium or urinary sodium, exposure to higher levels of PM_2.5_ was significantly associated with deteriorated lipid profiles. For example, each 10 μg/m^3^ incremental change in exposure to PM_2.5_ was correlated with a 2.83 (-4.65 to -0.97) lower percentage decrease in blood HDL-C levels among the participants who were from the highest quartile of urinary sodium levels. While, these associations changed to be nonsignificant in the participants who were from the lowest quartile of dietary sodium levels.

**Conclusion:**

Exposure to higher levels of PM_2.5_ was associated with deteriorated blood lipid levels in children and adolescents. It is noteworthy that these associations might be ameliorated through the adoption of a low-sodium dietary regimen.

**Supplementary Information:**

The online version contains supplementary material available at 10.1186/s12887-024-04896-8.

## Background

Atherosclerotic cardiovascular disease (ASCVD) is a well-documented risk for the global population. The Global Burden of Disease study provided evidence to indicate that approximately 32% of global deaths were attributable to prevalent ASCVDs [1]. The existing evidence supported the strong associations of abnormal blood lipids, such as too low levels of blood HDL-C, and high levels of blood LDL-C, with elevated risk of incident ASCVDs in adults [2, 3]. Recently, the blood lipid abnormalities have been increasingly reported in children and adolescents, with a worldwide incidence ranging from 10 to 30% [4–6]. Those with deteriorated lipid profiles in childhood and adolescence are at increased risk of having ASCVDs in their adulthood [7–9]. Therefore, identifying the risk factors associated with deteriorated lipid profiles in children and adolescents is essential to bring down the globally increasing morbidity and disease burden related to ASCVDs.

Exposure to higher levels of ambient PM_2.5_ has been widely documented as an important risk associated with deteriorated lipid profiles in adults [10–12]. However, the evidence in children and adolescents is limited. Mechanism evidence has emphasized the role of inflammatory response underlying the deteriorated effects of PM_2.5_ exposure on lipid profiles in the general population [13–15]. On the other hand, increasing evidence have documented the relationship between higher blood sodium levels with greater inflammation risks [16]. Meanwhile, the increasing rodent evidence have reported that a high-sodium exposure could induce the inflammation [17, 18]. Generally, the accumulation of sodium in human body is largely from the daily dietary consumptions [19]. Epidemiological evidence highlighted the association between a high-sodium diet and elevated inflammation risks [20, 21]. Therefore, a sodium-rich diet might have an role in inducing the individual-level inflammation responses. Since the pro-oxidant and pro-inflammatory responses are the key mechanisms linking PM_2.5_ exposure to dysregulation of lipid metabolism, it is plausible that a high-sodium diet could amplify the association of exposure to PM_2.5_ with deteriorated lipid profiles. However, population evidence which focused on examining the effect modification of a high-sodium diet on the associations between PM_2.5_ exposure and blood lipids, especially in children and adolescents, is still limited.

Therefore, we investigated the associations of exposure to PM_2.5_ with blood lipids in children and adolescents, and further examined the effect modifications of sodium levels on these associations based on a cross-sectional multi-community Chinese population.

## Methods

### Population and study design

We enrolled the participants aged from 6 to 17 years from a multi-community cross-sectional study, which were conducted between November 2015 and December 2017, covering 5 urban districts and 9 rural counties in Sichuan Province, China. In each region, two communities from urban districts or two towns from rural counties were selected using the systematic sampling (SS) method. In each community or town, one primary school and one middle school were selected by the simple random sampling (SRS) method. Limited by the population size and economic status, many communities or towns did not have senior high schools, therefore we did not randomly select senior high schools at the community or town level. Alternatively, we selected the study senior high schools at the district or county level by the SRS method. Finally, one class was randomly selected from each grade, and 28 students were SS selected from each class to participate in the study. A face-to-face interview was used to acquire information on the participants’ socio-economic and demographic characteristics, diet, physical activity, and health status. Anthropometric characteristics such as weight and height, and blood pressure were measured by physicians through standardized procedures.

Initially, 3784 participants were included. The participants missing the blood lipids measurements were excluded (*n* = 73). Initially, 3711 participants were included in the primary association analyses. Then, in the further stratified analyses, the participants missing dietary sodium information (*n* = 76), or urinary sodium information (*n* = 80) were also excluded, respectively. Finally, the stratified analysis using dietary sodium data and urinary sodium data included 3635 and 3631 participants, respectively. The details about the selection procedures were reported in the Supplementary Material Figure S1.

### Measurements of lipid outcomes

We took venous blood samples from all subjects. The plasma and serum samples were separated by centrifugation within 0.5–1.0 h and were dispensed into cryotubes according to a standardized protocol. Blood lipid biomarkers, including blood TC, blood HDL-C, blood LDL-C, and blood TG, were examined by experienced clinical laboratorians according to the standard operating procedures of the instruments in the laboratory of the study hospitals.

### Evaluation of PM_2.5_ exposures

Information on participants’ address for schools were obtained through a face-to-face interview. Annual average PM_2.5_ exposures, which covered the study areas, were assessed based on the global surface PM_2.5_ V4.CH.02 product of the Dalhousie University Atmospheric Composition Analysis Group (DUACAG) with a spatial resolution of 1 km x 1 km [22]. Briefly, the DUACAG applied a geographically weighted regression model combined with ground level PM_2.5_ monitors, remotely sensed aerosol optical depth (AOD), and multiangle imaging spectroradiometer (MISR) data, and outputs from the CEOS-Chem chemical transport model. Each participant’s school address was geocoded, and then the past 2-years’ average value of gridded PM_2.5_ data within a 1-km buffer area surrounding the school was calculated to indicate the long-term PM_2.5_ exposure for the study participants.

### Measurements of dietary sodium levels

A standardized semi-quantitative Food Frequency Questionnaire (FFQ) recommended by the Chinese CDC, which is a reliable and valid measurement for the Chinese population, was used to assess the individual-level dietary information [23]. The FFQ included dietary information on131 food items, which were specifically categorized into 28 food groups. Details of the frequently consumed food items of the FFQ were summarized in Supplementary Material Table S1. Information of each food group on the consumption frequency corresponding to the participants’ past 12 months was recorded as never, daily, weekly, monthly, or yearly for. Then, if the participants responded that they had consumed the specific food group, information on consumption amount for average intake per time was recorded by a Chinese unit of weight or capacity of Liang (50 g) or per cup (100 ml). We transformed all food consumption frequency into daily times by weekly times dividing 7, monthly times dividing 30, and yearly times dividing 365, respectively. Then, the daily amount of consumption for each food group was calculated by multiplying daily times and average intake amount per time. We estimated the daily dietary sodium intake according to the Chinese food composition database (2018), which was published by the Chinese CDC [24]. Additionally, we used the subjects’ urinary sodium levels as an internal biomarker to represent the dietary sodium levels. A random urine sample of 8–10 ml was collected from each study participant. Experienced clinical laboratorians examined the urinary sodium levels according to the standard operating procedures of the equipment in the study laboratory.

### Covariates

Participants’ socio-economic and demographic characteristics were collected, including residential region (urban, rural), as well as father’s ethnicity (minority, Han), education (0–6, 7–9, 10–12, and ≥ 13 years), and occupation (farmer/ retired worker, community/social service occupations, and skilled/professional/administrative jobs). The children’s demographic information, including age (years) and sex (male, female) were also collected. The body mass index (BMI), which was calculated by dividing height (kg) by the square of height (meters), was used to represent a participant's physical fitness. Behavioral characteristics that could potentially affect children, such as passive smoking status (no, yes), and daily outdoor activity duration (hours), were collected.

### Statistical analysis

We summarized the distribution of demographic, social-economic, and behavioral characteristics of the study participants across urinary sodium strata by count and proportion. The results of significant tests for these characteristics across urinary sodium strata were estimated by chi-square tests or Fisher exact tests.

Linear regression models were applied to explore the correlations of per 10 ug/m^3^ increase in PM_2.5_ exposure with blood lipid outcomes. We further categorized the PM_2.5_ exposure into quartiles, and estimated the associations of the 2rd to highest quartiles of PM_2.5_ exposure with blood lipids, compared with the lowest PM_2.5_ exposure, to examine the potential dose–response relationships of the associations. The results of “*P* for trend” for blood lipid outcomes associated with PM_2.5_ quartiles were calculated by modeling the median values of PM_2.5_ within each quartile stratum as a continuous variable. Additionally, we examined the potential non-linear associations of PM_2.5_ exposure with blood lipids using the restricted cubic spline methods. In the multivariable-adjusted analysis, a direct acyclic graphic (DAG) method was introduced to determine the potential confounders (Supplementary Material Figure S2). To assess the effect modification attributed to dietary sodium levels on the associations of PM_2.5_ exposure with blood lipid outcomes, we conducted stratified analyses by dietary sodium quartiles and urinary sodium quartiles. In each stratum, a linear regression model which adjusted the covariates in the DAG diagram was fitted in order to explore the study associations. To obtain an overall assessment of the effect modification, we applied the log-likelihood ratio test by comparing the difference between the model with the interaction term and the model without that interaction term. We performed a number of sensitivity analyses to evaluate the robustness of the study associations. We first evaluated the dietary variations on the study associations by additionally adjusting for the dietary intake of total fat and total energy. In addition, we excluded the participants whose parents have been diagnosed with cardiovascular diseases to evaluate the genetic variations on the study associations. Finally, we conducted a sensitivity analysis by replicating the stratified analyses based on creatinine-adjusted urinary sodium concentrations, to allow for the random urine samples’ variation in dilution.

All statistical analyses in this study were performed using SAS 9.4. The significance level was determined by a two-sided test with a “*P* value < 0.05”.

## Results

Table 1 summarized the distribution of demographic, social-economic, and behavioral characteristics for the study participants across urinary sodium quartiles. The participants who were from rural areas, had fathers belonging to ethnical minorities or accepting poor education, or being farmers/retirees, were more likely to have higher urinary sodium levels (Table 1). The children who had a higher BMI and were older preferred a high-sodium diet. The distribution of long-term PM_2.5_ exposure levels ranged from 22.4 to 65.0 μg/m^3^ among the study participants (Table 1). Those exposed to the highest quartiles of PM_2.5_ exposure had higher levels of blood LDL-C (Table 2).

**Table 1:** The demographic and socio-economic characteristics for study participants stratified by urinary sodium levels. (*N* = 3631)

Covariates	Childhood urinary sodium levels, quartile				*P*- value
	Quartile 1	Quartile 2	Quartile 3	Quartile 4	
**Region, n (%)**					＜0.001
Urban	359 (27.32)	371 (28.23)	338 (25.72)	246 (18.72)	
Rural	549 (23.69)	541 (23.35)	576 (24.86)	651 (28.10)	
**Father’s ethnicity, n(%)**					＜0.001
Minority	29 (13.06)	32 (14.41)	75 (33.78)	86 (38.74)	
Han	879 (25.78)	880 (25.81)	839 (24.61)	811 (23.79)	
**Father's educational years, n (%)**					＜0.001
0~6	206 (22.06)	183 (19.59)	254 (27.19)	291 (31.16)	
7~9	399 (23.71)	434 (25.79)	419 (24.90)	431 (25.61)	
10~12	175 (27.47)	187 (29.36)	149 (23.39)	126 (19.78)	
≥13	128 (34.04)	107 (28.46)	92 (24.47)	49 (13.03)	
**Father's occupation, n (%)**					＜0.001
Farmer/retired	141 (22.93)	136 (22.11)	161 (26.18)	177 (28.78)	
Community/social services occupations	629 (24.35)	666 (25.78)	648 (25.09)	640 (24.78)	
Skilled/professional/administration occupations	138 (32.09)	109 (25.35)	105 (24.42)	78 (18.14)	
**Child BMI, kg/m**^2^**, median(IQR)**	16.36 (14.83- 18.46)	16.54 (15.02- 18.76)	17.22 (15.30- 19.64)	18.01 (15.85- 20.39)	＜0.001
**Child's age, median (IQR)**	10 (8- 13)	10 (8- 13)	11 (9- 13)	12 (10- 15)	＜0.001
**Child sex, n (%)**					＜0.001
Male	349 (19.06)	442 (24.14)	520 (28.40)	520 (28.40)	
Female	559 (31.06)	470 (26.11)	394 (21.89)	377 (20.94)	
**Daily outdoor activity duration, hours, median (IQR)**	0.29 (0.11- 0.64)	0.29 (0.14- 0.71)	0.29 (0.14- 0.71)	0.29 (0.11- 0.64)	0.468
**Passive smoking, n (%)**					0.375
No	486 (25.19)	484 (25.09)	466 (24.16)	493 (25.56)	
Yes	422 (24.91)	428 (25.27)	445 (26.27)	399 (23.55)

**Table 2:** The distributions of blood lipid outcomes in children and adolescents stratified by PM_2.5_ exposures. (*N* = 3711)

Blood lipids, mmol/L	PM_2.5_ exposures, μg/m^3^				*P*-value
Median (Q1-Q3)	Quartile 1 (22.4-42.8)	Quartile 2 (42.9-54.4)	Quartile 3 (54.5-59.0)	Quartile 4 (59.1-65.0)	
TC	3.58 (3.24- 3.98)	3.68 (3.34- 4.11)	3.72 (3.33- 4.15)	3.72 (3.35- 4.14)	< 0.0001
HDL-C	1.53 (1.31- 1.76)	1.53 (1.31- 1.80)	1.47 (1.27- 1.70)	1.52 (1.31- 1.75)	< 0.0001
LDL-C	1.84 (1.58- 2.15)	1.98 (1.67- 2.31)	1.93 (1.62- 2.32)	2.03 (1.68- 2.38)	< 0.0001
TG	0.77 (0.62- 1.00)	0.79 (0.61- 1.04)	0.80 (0.62- 1.06)	0.78 (0.63- 0.99)	0.0704

The results of association analyses between PM_2.5_ exposure and blood lipids were presented in Table 3. After adjusted for confounders, per 10 μg/m^3^ incremental change in PM_2.5_ was related to a 1.56% (95% confidence interval 0.90%—2.23%), 2.26% (1.15—3.38) higher level of blood TC and LDL-C, and a 1.49% (-2.35% to -0.62%) lower level of blood HDL-C. Similar patterns persisted when PM_2.5_ exposures were modeled as changes in quartiles with blood TC, and LDL-C levels. The results of restricted cubic spline regression analyses did not support non-linear associations of PM_2.5_ exposure with blood lipids (Supplementary Figure S3). No associations were found between long-term exposure to PM_2.5_ and blood TG.

**Table 3:** The associations of exposure to PM_2.5_ with blood lipid outcomes in children and adolescents.^a^ (*N* = 3711)

PM_2.5_ exposures	Adjusted percentage changes (95% CI)			
	TC	HDL-C	LDL-C	TG
Per 10 μg/m^3^	1.56 (0.90, 2.23)*	-1.49 (-2.35, -0.62)*	2.26 (1.15, 3.38)*	0.08 (-1.43, 1.62)
Quartiles, quartile 1	Reference	Reference	Reference	Reference
Quartile 2	3.20 (1.50, 4.92)*	0.75 (-1.47, 3.03)	4.97 (2.11, 7.91)*	-0.16 (-3.94, 3.78)
Quartile 3	4.43 (2.73, 6.15)*	-3.32 (-5.43, -1.16)*	5.39 (2.56, 8.30)*	3.93 (0.04, 7.97)
Quartile 4	4.46 (2.52, 6.43)*	-1.00 (-3.47, 1.53)	5.95 (2.70, 9.30)*	-1.07 (-5.29, 3.34)
*P* for trend	< 0.0001	0.0044	< 0.0001	0.8323

^*^P < 0.05

^a ^Adjusted for region, father’s ethnicity, father’s occupation, father’s education, child’s age, child’s sex, child’s BMI, childhood daily average outdoor physical activity duration and passive smoking

**Fig. 1:**
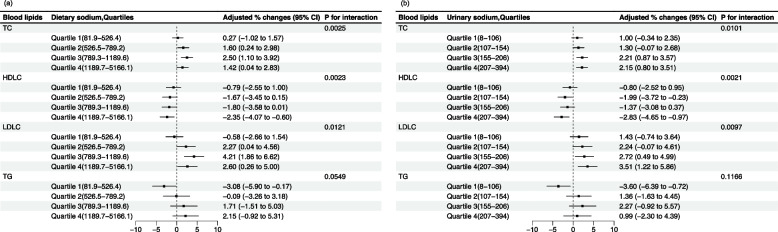
The associations of PM_2.5_ with blood lipids stratified by: (**a**) dietary sodium(*N* = 3635), (**b**) urinary sodium (*N* = 3631)

We examined the effect modification by dietary sodium intake and urinary sodium excretion on the relationships between exposure to PM_2.5_ and blood lipid outcomes. The results of the effect modification analyses by dietary sodium and urinary sodium are shown in Fig. 1 a, b, and Supplementary Material Table S2. We found strong evidence to indicate that a high-sodium diet modified the study associations (*P* for interaction < 0.05). For the participants who were in the highest dietary solidum strata, per 10 μg/m^3^ increase in PM_2.5_ exposure was related to a 1.42 (95% *CI* 0.04 to 2.83) higher percentage increase in blood TC levels, a 2.60 (95% *CI* 0.26 to 5.00) higher percentage increase in blood LDL-C levels, and a 2.35 (95% *CI* -4.07 to -0.60) lower percentage decrease in blood HDL-C levels. However, these relationships between PM_2.5_ exposure and deteriorated lipid profiles changed to non-significant among the participants who were from the lowest quartile of dietary sodium levels. The similar associations were observed in replicating the main analyses by different urinary sodium quartiles, which yield pronounced associations of exposure to higher PM_2.5_ levels with deteriorated blood TC, HDL-C, and LDL-C in the participants who were from the highest urinary sodium quartile strata. The associations changed to be nonsignificant in the participants who were from the lowest quartile level of urinary sodium. Although the stratified analyses documented negative associations of PM_2.5_ exposure with blood TG levels among the participants from the lowest quartile of sodium level, the results of significant tests did not support the difference (*P* for interaction > 0.05).

**Fig. 2:**
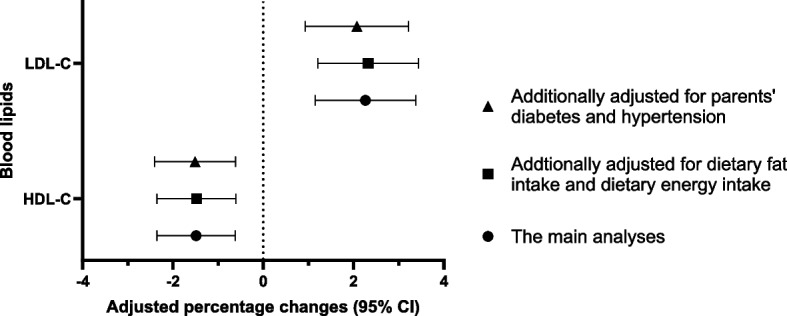
Results of sensitivity analyses for PM_2.5_ and lipids^a^. **a** The main analyses (*N*=3711), additionally adjusted for dietary fat intake and dietary energy intake (*N*=3711), and additionally adjusted for parents’ diabetes and hypertension prevalence (*N*=3475)

In the sensitivity analyses, when dietary fat intake and dietary energy intake were included in the model, the results remained little changes (Fig. 2 and Supplementary Material Table S3). When excluded the participants whose parents had diabetes or hypertension, there were also little changes in the results (Fig. 2 and Supplementary Material Table S4). When we considered the variations caused by urinary creatinine, the results of association analyses of PM_2.5_ with blood lipids stratified by creatinine-adjusted urinary sodium levels were similar to the main results (Supplementary Material Figure S4).

## Discussion

By employing a cross-sectional data with multi-community population, we explored the relationships of long-term PM_2.5_ exposure with blood lipids in children and adolescents. The results showed that exposure to higher levels of ambient PM_2.5_ was related to higher blood TG, LDL-C, and lower HDL-C levels. Most importantly, we provided consistent evidence to support the effect modification of a high-sodium diet on the association between PM_2.5_ exposure and blood lipids in children and adolescents.

Our findings that PM_2.5_ exposure may lead to the deteriorated lipid profiles were consistent with existing evidence. A study in Iran involving 1413 adolescents found that air quality index was positively correlated with blood TC, blood LDL-C and blood TG, and negatively correlated with blood HDL-C. [25]. Another Iranian study which involved 186 children and adolescents from 10 to 18 years of age showed positive correlations between PM_2.5_ exposure levels and blood LDL-C or TG levels [26]. Similarly, a cross-sectional survey of China involving 12,814 children aged from 7 to 18, showed that the children and adolescents who were chronically exposure to PM_2.5_, had higher blood TC levels [14]. In our study, featured by a multi-ethnic Chinese population, we also demonstrated that there were associations between exposure to higher levels of ambient PM_2.5_ surrounding school and deteriorated lipid profiles, including higher blood TC, higher blood LDL-C, and lower blood HDL-C levels in children and adolescents,. Our study highlighted the importance of reducing the school-level PM_2.5_ exposure on benefit blood lipids in children and adolescents.

Although several studies have highlighted the modification of diet on the associations of air pollution situation and prevalence and mortality of cardiovascular disease in adults and elderly [27, 28], few studies have examined the role of dietary sodium underlying the association between atmospheric pollutants and blood lipids in children and adolescents. In this study, based on dietary estimates and urinary biomarkers, we provided consistent evidence that a lower sodium diet could reduce the toxic association of PM_2.5_ exposure on blood lipids in children and adolescents.

The modifcations of sodium levels on the association between PM_2.5_ and blood lipids can be explained by several mechanisms. It is well known that a high-sodium diet could induce the oxidative stress responses and inflammatory that lead to increased risk of cardiovascular diseases [24]. A number of studies have explained that exposure to high concentrations of PM_2.5_ may disrupt lipid metabolism through pro-oxidative and pro-inflammatory effects [29–31]. Therefore, we can speculate that a lower sodium diet modulated the correlations between PM_2.5_ exposure and blood lipids by reducing the PM_2.5_ exposure related pro-oxidative and pro-inflammatory effects. Since the important role of abnormal lipid levels in childhood in predicting the subsequent risks of incident ASCVDs in adulthood, our findings provide a new insight to treat the low-sodium diet as a potential preventive method to reduce the risk of incident ASCVDs attributed to PM_2.5_ exposure in children and adolescents.

There are some strengths and limitations in our study. We explored the relationships between long-term PM_2.5_ exposure and blood lipid outcomes in children and adolescents based on 3711 participants who provided measurements of four blood lipid outcomes. We adjusted for many confounders such as family-socio-economic differences, passive smoking status, child’s BMI, and outdoor physical activity. We estimated the effect modification of dietary sodium on the study associations using semi-quantitative FFQ methods and urinary internal biomarkers related to sodium consumption, and the results indicated strong dose–response patterns. However, there are still several limitations that need to be declared. First, the nature of cross-sectional study prevented us from establishing causal relationships for the study associations. Second, limited by the data availability, we only evaluated the long-term exposure of PM_2.5_ based on schools, which missed the exposures from residence. In addition, a family's cooking and eating habits may affect a child's dietary sodium intake, and we did not collected these information, which may influenced the conclusions.

## Conclusions

Exposure to higher levels of PM_2.5_ is associated with deteriorated lipid profiles in children and adolescents. These associations became null in the participants who maintained a low-sodium diet. A low dietary sodium diet could mitigate the PM_2.5_ related damage on blood lipids in children and adolescents.

### Supplementary Information

Supplementary Material 1.

Supplementary Material 2.

## Data Availability

The datasets used and/or analyzed during the current study are available from the corresponding author upon reasonable request.

## Abbreviations

PM_2.5_: Fine particulate matter
TC: Total cholesterol
HDL-C: High-density lipoprotein cholesterol
LDL-C: Low-density lipoprotein cholesterol
TG: Triglycerides
FFQ: Food frequency questionnaire
ASCVDs: Atherosclerotic cardiovascular diseases
SS: Systematic sampling
SRS: Simple random sampling
CDC: Center for Disease Control and Prevention
DUACAG: Dalhousie University Atmospheric Composition Analysis Group
AOD: Aerosol optical depth
MISR: Multiangle imaging spectroradiometer
BMI: Body mass index
DAG: Direct acyclic graphic

## References

[1] Word Health Organization. Fact Sheets. Cardiovascular Diseases(CVDs). 2021. https://www.who.int/news-room/fact-sheets/detail/cardiovascular-diseases-(cvds). Accessed 20 Nov 2023.

[2] Ference BA, Graham I, Tokgozoglu L, Catapano AL. Impact of lipids on cardiovascular health: JACC Health Promotion Series. J Am Coll Cardiol. 2018;72(10):1141–56. 30165986 10.1016/j.jacc.2018.06.046

[3] Jellinger PS, Handelsman Y, Rosenblit PD, et al. American association of clinical endocrinologists and american college of endocrinology guidelines for management of dyslipidemia and prevention of cardiovascular disease. Endocr Pract. 2017;23(Suppl 2):1–87. 28437620 10.4158/EP171764.APPGL

[4] Costa-Urrutia P, Colistro V, Franco-Trecu V, Granados J, Álvarez Fariña R, Rodríguez-Arellano ME. Dyslipidemia, obesity, and ethnicity in mexican children. Int J Environ Res Public Health. 2021;18(23):12659. 34886385 10.3390/ijerph182312659 PMC8656470

[5] Gomes ÉIL, Zago VHS, Faria EC. Evaluation of lipid profiles of children and youth from basic health units in campinas, SP, Brazil: a cross-sectional laboratory study. Arq Bras Cardiol. 2020;114(1):47–56. 31644697 10.5935/abc.20190209 PMC7025304

[6] Stone NJ, Smith SC Jr, Orringer CE, et al. Managing atherosclerotic cardiovascular risk in young adults: JACC state-of-the-art review. J Am Coll Cardiol. 2022;79(8):819–36. 35210038 10.1016/j.jacc.2021.12.016

[7] Berenson GS, Srinivasan SR, Bao W, Newman WP 3rd, Tracy RE, Wattigney WA. Association between multiple cardiovascular risk factors and atherosclerosis in children and young adults. The Bogalusa Heart Study. N Engl J Med. 1998;338(23):1650–6. 9614255 10.1056/NEJM199806043382302

[8] Perak AM, Ning H, Khan SS, et al. Associations of late adolescent or young adult cardiovascular health with premature cardiovascular disease and mortality. J Am Coll Cardiol. 2020;76(23):2695–707. 33181243 10.1016/j.jacc.2020.10.002 PMC8100998

[9] Domanski MJ, Tian X, Wu CO, et al. Time course of ldl cholesterol exposure and cardiovascular disease event risk. J Am Coll Cardiol. 2020;76(13):1507–16. 32972526 10.1016/j.jacc.2020.07.059

[10] Zhang K, Wang H, He W, et al. The association between ambient air pollution and blood lipids: a longitudinal study in Shijiazhuang. China Sci Total Environ. 2021;752: 141648. 32889259 10.1016/j.scitotenv.2020.141648

[11] Gui ZH, Yang BY, Zou ZY, et al. Exposure to ambient air pollution and blood lipids in children and adolescents: a national population based study in China. Environ Pollut. 2020;266(Pt 3): 115422. 32829032 10.1016/j.envpol.2020.115422

[12] McGuinn LA, Schneider A, McGarrah RW, et al. Association of long-term PM2.5 exposure with traditional and novel lipid measures related to cardiovascular disease risk. Environ Int. 2019;122:193–200. 30446244 10.1016/j.envint.2018.11.001 PMC6467069

[13] Bai Y, Sun Q. Fine particulate matter air pollution and atherosclerosis: Mechanistic insights. Biochim Biophys Acta. 2016;1860(12):2863–8. 27156486 10.1016/j.bbagen.2016.04.030

[14] Brook RD, Rajagopalan S, Pope CA 3rd, et al. Particulate matter air pollution and cardiovascular disease: an update to the scientific statement from the American Heart Association. Circulation. 2010;121(21):2331–78. 20458016 10.1161/CIR.0b013e3181dbece1

[15] Wan Q, Cui X, Shao J, et al. Beijing ambient particle exposure accelerates atherosclerosis in ApoE knockout mice by upregulating visfatin expression. Cell Stress Chaperones. 2014;19(5):715–24. 24523034 10.1007/s12192-014-0499-2 PMC4147068

[16] Patel S, Rauf A, Khan H, Abu-Izneid T. Renin-angiotensin-aldosterone (RAAS): The ubiquitous system for homeostasis and pathologies. Biomed Pharmacother. 2017;94:317–25. 28772209 10.1016/j.biopha.2017.07.091

[17] Uetake Y, Ikeda H, Irie R, et al. High-salt in addition to high-fat diet may enhance inflammation and fibrosis in liver steatosis induced by oxidative stress and dyslipidemia in mice. Lipids Health Dis. 2015;14:6. 25888871 10.1186/s12944-015-0002-9 PMC4337194

[18] Huang P, Shen Z, Yu W, et al. Hydrogen sulfide inhibits high-salt diet-induced myocardial oxidative stress and myocardial hypertrophy in dahl rats. Front Pharmacol. 2017;8:128. 28360857 10.3389/fphar.2017.00128 PMC5352693

[19] Fang K, He Y, Fang Y, Lian Y. Dietary sodium intake and food sources among chinese adults: Data from the CNNHS 2010–2012. Nutrients. 2020;12(2):453. 32054013 10.3390/nu12020453 PMC7071264

[20] Zhu H, Pollock NK, Kotak I, et al. Dietary sodium, adiposity, and inflammation in healthy adolescents. Pediatrics. 2014;133(3):e635–42. 24488738 10.1542/peds.2013-1794 PMC3934330

[21] Robinson AT, Edwards DG, Farquhar WB. The Influence of dietary salt beyond blood pressure. Curr Hypertens Rep. 2019;21(6):42. 31025198 10.1007/s11906-019-0948-5 PMC7309298

[22] van Donkelaar A, Martin RV, Brauer M, et al. Global estimates of fine particulate matter using a combined geophysical-statistical method with information from satellites, models, and monitors. Environ Sci Technol. 2016;50(7):3762–72. 26953851 10.1021/acs.est.5b05833

[23] Zhao W-H, Huang Z-P, Zhang X, et al. Reproducibility and validity of a chinese food frequency questionnaire. Biomed Environ Sci. 2010;23:1–38.20486429 10.1016/S0895-3988(11)60014-7

[24] Yang YX. China Food Composition. Standard. Beijing: Peking University Medical Press; 2018.

[25] Poursafa P, Mansourian M, Motlagh ME, Ardalan G, Kelishadi R. Is air quality index associated with cardiometabolic risk factors in adolescents? The CASPIAN-III Study Environ Res. 2014;134:105–9. 25127520 10.1016/j.envres.2014.07.010

[26] Poursafa P, Amin MM, Mansourian M, et al. Association of exposure to fine particulate matter and risk factors of non-communicable diseases in children and adolescents. Int J Pediatr. 2017;5:5871–80.

[27] Lim CC, Hayes RB, Ahn J, et al. Mediterranean diet and the association between air pollution and cardiovascular disease mortality risk. Circulation. 2019;139(15):1766–75. 30700142 10.1161/CIRCULATIONAHA.118.035742 PMC6453737

[28] Wang M, Zhou T, Song Q, et al. Ambient air pollution, healthy diet and vegetable intakes, and mortality: a prospective UK Biobank study. Int J Epidemiol. 2022;51(4):1243–53. 35179602 10.1093/ije/dyac022 PMC9365625

[29] Tian N, Moore RS, Braddy S, et al. Interactions between oxidative stress and inflammation in salt-sensitive hypertension. Am J Physiol Heart Circ Physiol. 2007;293(6):H3388–95. 17921322 10.1152/ajpheart.00981.2007

[30] Long MH, Zhu XM, Wang Q, et al. PM2.5 exposure induces vascular dysfunction via NO generated by iNOS in lung of ApoE-/- mouse. Int J Biol Sci. 2020;16(1):49–60. 31892845 10.7150/ijbs.36073 PMC6930374

[31] Sørensen M, Daneshvar B, Hansen M, et al. Personal PM2.5 exposure and markers of oxidative stress in blood. Environ Health Perspect. 2003;111(2):161–6. 12573899 10.1289/ehp.111-1241344 PMC1241344

