# Supplementary material for: Moderating effect of a sodium-rich diet on the association between long-term exposure to fine particulate matter and blood lipids in children and adolescents
Source: BMC Pediatr. 2024 Jul 20;24:466. doi: 10.1186/s12887-024-04896-8 (PMC11264876; doi:10.1186/s12887-024-04896-8)
Supplement: Supplementary file 1 — Supplementary Material 1. [file 12887_2024_4896_MOESM1_ESM.docx]

**Supplementary Tables and Figures**


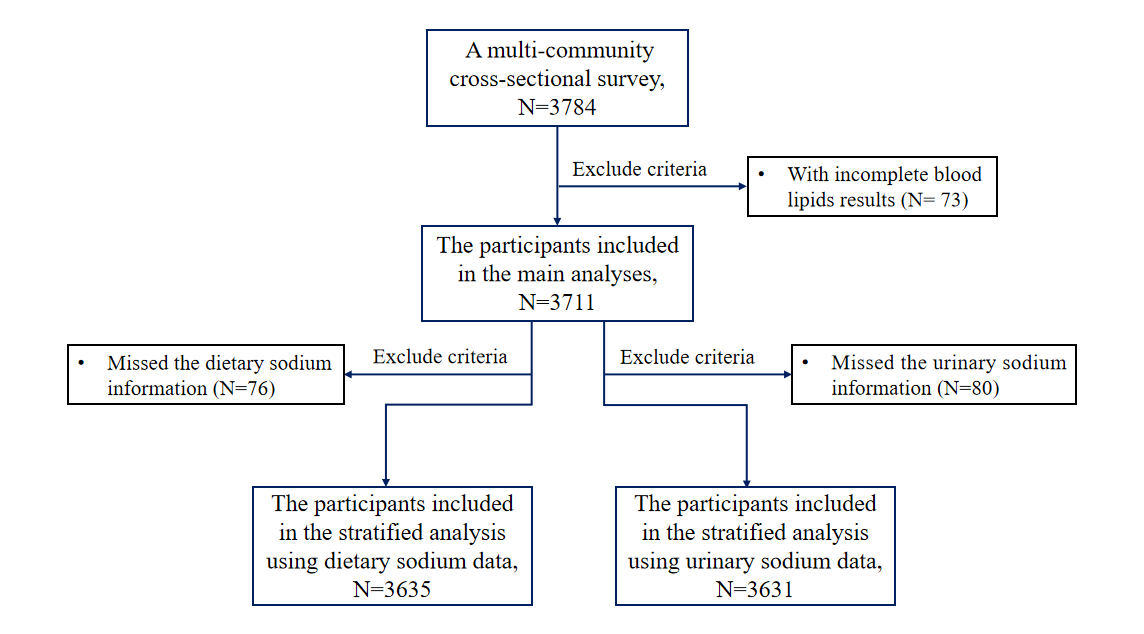


**Supplementary Figure S1.** The inclusion and exclusion flow chart.


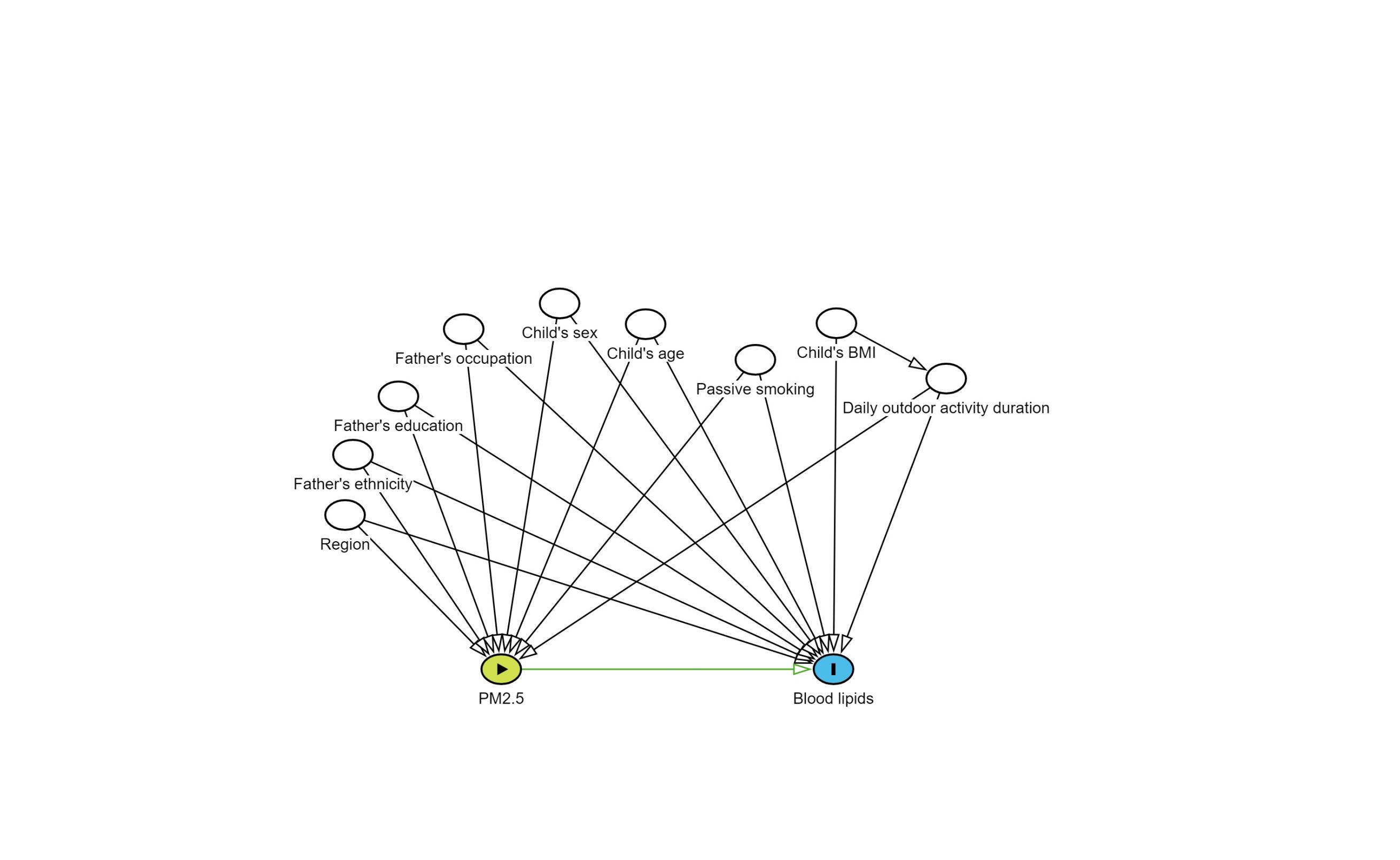


**Supplementary Figure S2.** The fully DAG-based confounders in multivariable-adjusted regression models between exposure to PM_2.5_ and blood lipids.

**
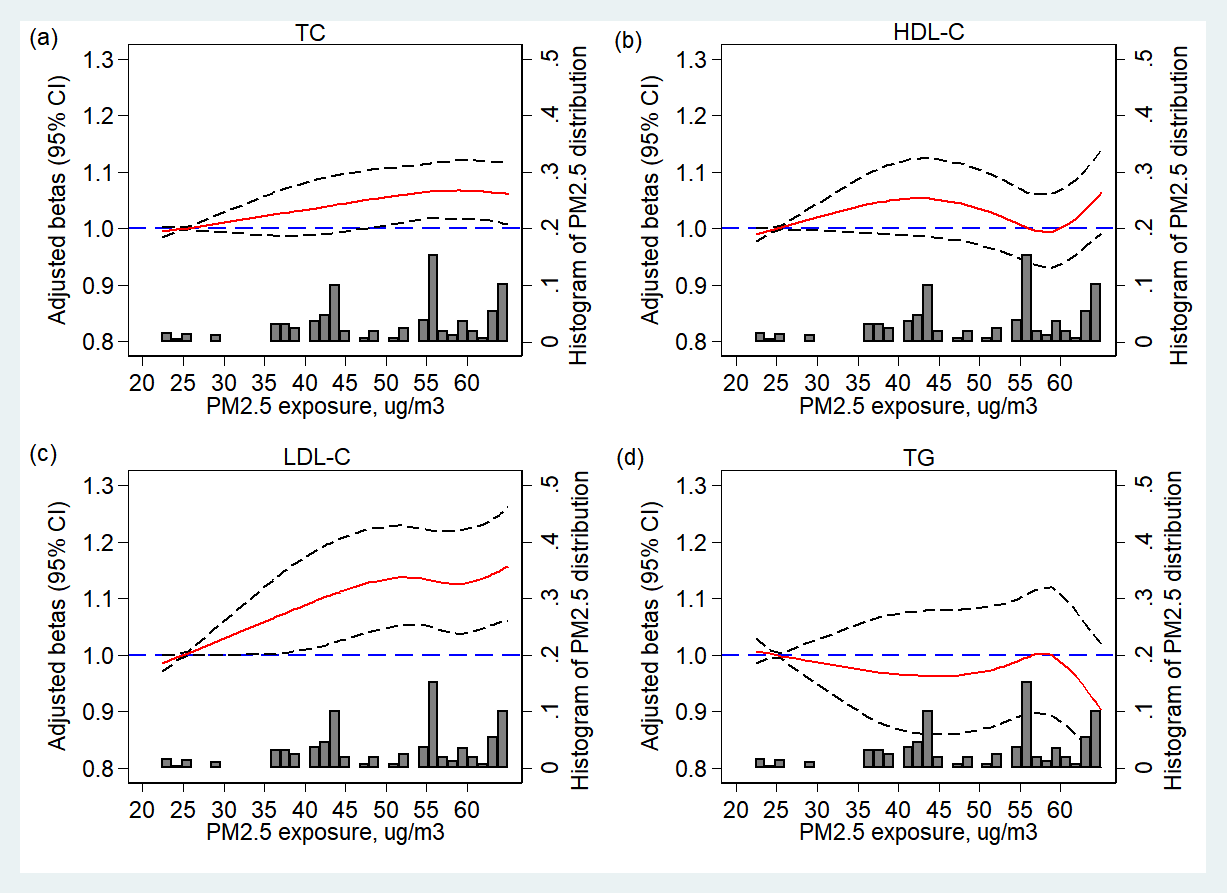
**

Supplementary Figure S3. Restricted cubic spline (RCS) analysis of the associations between PM_2.5_ exposure and blood lipids.


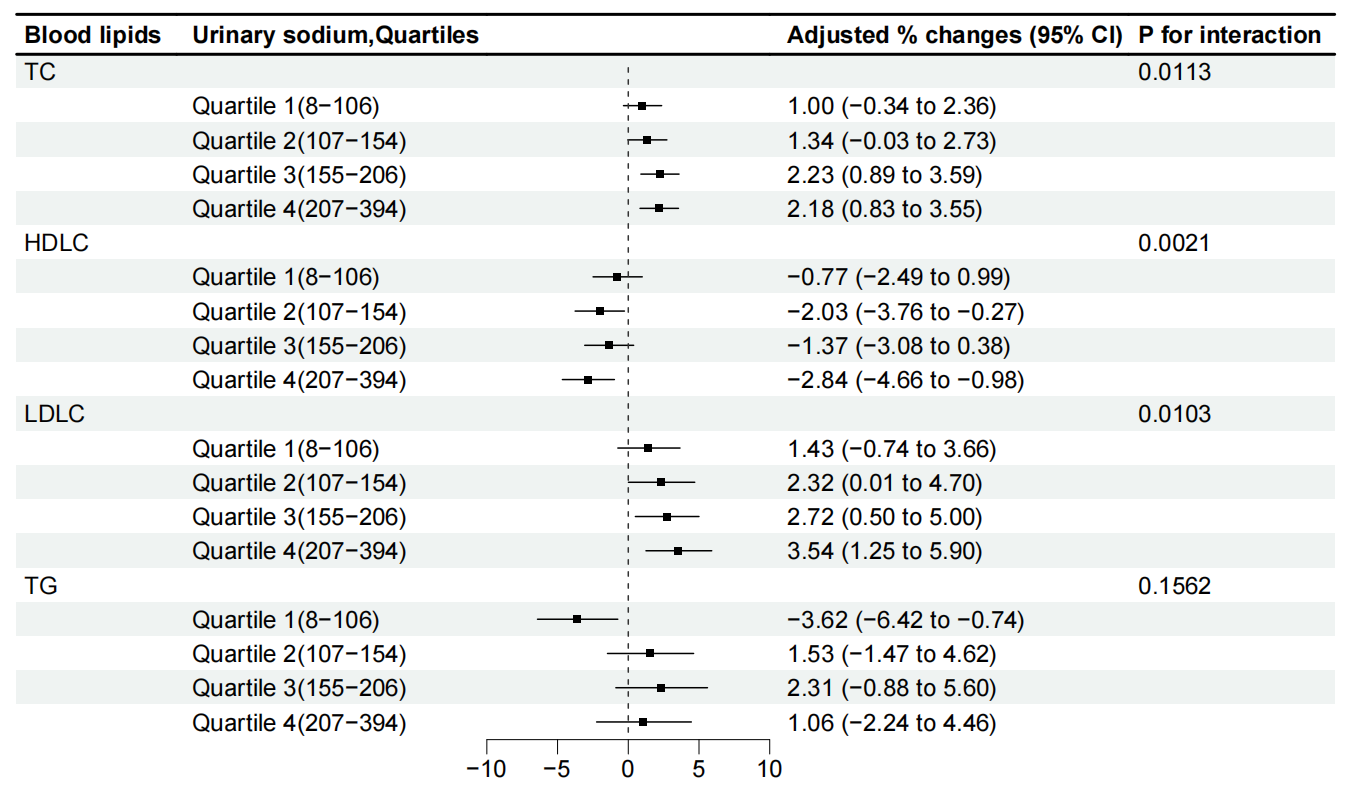


Supplementary Figure S4. The associations of PM_2.5_ with blood lipids stratified by creatinine-adjusted urinary sodium. (N=3631)

**Supplementary Table S1**. Study participants’ specific common consumptions of food items under food groups listed in FFQ.

| **Serial number** | **Food groups in FFQ** | **Unit in FFQ** | **Food items** |
| --- | --- | --- | --- |
| 1 | [Cereal](javascript:;) | gram | Rice, Rice noodles, Steamed bread, Noodles, Corn products, Millet, Buckwheat |
| 2 | Tuber crops | gram | Potatoes, Red sweet potatoes, White sweet potatoes |
| 3 | Mixed beans | gram | Mung beans, Red beans, Pinto beans |
| 4 | Fried dough foods | gram | Fried dough sticks, Fried bread, Instant noodles |
| 5 | Beans | gram | Soy beans, Black beans, Green beans, Soy milk, Soybean curd, Tofu, Tofu bamboo, Shredded tofu, Dried tofu |
| 6 | Vegetables | gram | Chinese cabbage, Asparagus lettuce, Cauliflower, Cucumber, Eggplant, Tomato, Brassica chinensis, Water spinach, Winter melon, Lotus root, Spinach, Pumpkin, Cabbage, Radish |
| 7 | Dried vegetable | gram | Chinese cabbage(dehydrated), Rape (dehydrated) , Spinach (dehydrated) |
| 8 | Pickled vegetables | gram | Pickles, Sauerkraut |
| 9 | Phytocomycetes | gram | Mushroom, Agaric fungus, Tremella fungus, Bamboo fungus, Kelp |
| 10 | Fruit | gram | Apple, Banana, Grape, Watermelon, Orange, Pear, Pomelo, Peach, Mango, Dragon fruit, Longan, Kiwi, Plum, Strawberry, Pineapple, Lychee, Date, Star fruit |
| 11 | Pig pork | gram | Pig pork |
| 12 | Red meats | gram | Beef, Mutton, Donkey, Horse, Rabbit, Dog meat |
| 13 | Animal meats | gram | Chicken, Duck, Goose, Pigeon |
| 14 | Meat product | gram | Luncheon meat, Ham sausage, Sausage |
| 15 | Animal blood | gram | Animal blood |
| 16 | Animal liver | gram | Animal liver |
| 17 | Aquatic products | gram | Freshwater fish, Sea fish, Shrimp, Crabs, Mollusks |
| 18 | Egg | gram | Eggs, Duck eggs, Quail eggs, Goose eggs, Century eggs, Salted egg |
| 19 | Milk products | gram | Milk, Milk powder, Yogurt, Cheese, Milk curd |
| 20 | Seeds | gram | Peanut |
| 21 | Nuts | gram | Walnuts, Chestnuts, Pistachios, Cashews, Sunflower seeds |
| 22 | Dried fruit | gram | Dried dates, Dried apricots, Raisins, Dried persimmon |
| 23 | Snacks | gram | Puffed food, Preserves, Chocolate, Candy, Ice cream |
| 24 | Juice | gram | 100% fruit and vegetable juice, not 100% fruit and vegetable juice |
| 25 | Carbonate beverages | gram | Cola, Fanta, Sprite, Soda water |
| 26 | Beverages | gram | Tea drinks, Dairy drinks, Vegetable protein and cereal drinks, Energy drinks, Other sugary drinks |
| 27 | Coffee | gram | Instant, bottled, freshly ground coffee |
| 28 | Cake | gram | Cake, Bread, Cookie |

**Supplementary Table S2**. The associations of exposure to PM_2.5_ with blood lipids stratified by dietary and urinary sodium.

| **Exposures/outcomes** | **Adjusted % changes(95% CI)** | | | | ***P* for interaction** |
| --- | --- | --- | --- | --- | --- |
|  | **Quartile 1** | **Quartile 2** | **Quartile 3** | **Quartile 4** |  |
| **Dietary sodium**  **(N=3635)** |  |  |  |  |  |
| TC | 0.27(-1.02, 1.57) | 1.60(0.24, 2.98) | 2.50(1.10, 3.92) | 1.42(0.04, 2.83) | 0.0025 |
| HDL-C | -0.79(-2.55, 1.00) | -1.67(-3.45, 0.15) | -1.80(-3.58, 0.01) | -2.35(-4.07, -0.60) | 0.0023 |
| LDL-C | -0.58(-2.66, 1.54) | 2.27(0.04, 4.56) | 4.21(1.86, 6.62) | 2.60(0.26, 5.00) | 0.0121 |
| TG | -3.08(-5.90, -0.17) | -0.09(-3.26, 3.18) | 1.71(-1.51, 5.03) | 2.15(-0.92, 5.31) | 0.0549 |
| **Urinary sodium**  **(N=3631)** |  |  |  |  |  |
| TC | 1.00(-0.34, 2.35) | 1.30( -0.07, 2.68) | 2.21(0.87, 3.57) | 2.15(0.80, 3.51) | 0.0101 |
| HDL-C | -0.80(-2.52, 0.95) | -1.99(-3.72, -0.23) | -1.37(-3.08, 0.37) | -2.83(-4.65, -0.97) | 0.0021 |
| LDL-C | 1.43(-0.74, 3.64) | 2.24(-0.07, 4.61) | 2.72(0.49, 4.99) | 3.51(1.22, 5.86) | 0.0097 |
| TG | -3.60(-6.39, -0.72) | 1.36(-1.63, 4.45) | 2.27(-0.92, 5.57) | 0.99(-2.30, 4.39) | 0.1166 |

**Supplementary Table S3**. Results of sensitivity analysis when dietary fat intake and dietary energy intake were included.^a^ (N=3711)

| **PM_2.5_ exposures** | **Adjusted percentage changes (95% CI)** | | | |
| --- | --- | --- | --- | --- |
|  | **TC** | **HDL-C** | **LDL-C** | **TG** |
| Per 10 μg/m^3^ | 1.59(0.92, 2.26)* | -1.48(-2.35, -0.60)* | 2.32(1.21, 3.44)* | 0.06(-1.46, 1.60) |
| Quartiles, quartile 1 | Reference | Reference | Reference | Reference |
| Quartile 2 | 3.19(1.49, 4.92)* | 0.71(-1.52, 2.99) | 4.95(2.08, 7.89)* | 0.07(-3.74, 4.02) |
| Quartile 3 | 4.51(2.8, 6.24)* | -3.33(-5.45, -1.17)* | 5.56(2.71, 8.49)* | 4.08(0.17, 8.15) |
| Quartile 4 | 4.58(2.64, 6.57)* | -0.97(-3.45, 1.56) | 6.22(2.96, 9.59)* | -1.10(-5.33, 3.32) |
| *P* for trend | ＜0.0001 | 0.0051 | ＜0.0001 | 0.8506 |

**P* < 0.05.

a. Adjusted for region, father’s ethnicity, father’s occupation, father’s education, child’s age, child’s sex, child’s BMI, childhood daily average outdoor physical activity durations, passive smoking, dietary fat and dietary energy.

**Supplementary Table S4**. Results of sensitivity analysis when parents had diabetes or hypertension were excluded.^a^ (N=3475)

| **PM_2.5_ exposures** | **Adjusted percentage changes (95% CI)** | | | |
| --- | --- | --- | --- | --- |
|  | TC | HDL-C | LDL-C | TG |
| Per 10 μg/m^3^ | 1.44(0.75,2.13)* | -1.51(-2.41,-0.61)* | 2.07(0.93,3.22)* | 0.03(-1.53,1.61) |
| Quartiles, quartile 1 | Reference | Reference | Reference | Reference |
| Quartile 2 | 3.08(1.34,4.86)* | 0.48(-1.8,2.82) | 4.7(1.79,7.71)* | 0.26(-3.63,4.31) |
| Quartile 3 | 4.05(2.31,5.82)* | -3.17(-5.35, -0.94)* | 4.6(1.72,7.57)* | 3.55(-0.43,7.69) |
| Quartile 4 | 4.35(2.36,6.39)* | -1.38(-3.91,1.22) | 5.76(2.44,9.19)* | -0.14(-4.51,4.43) |
| *P* for trend | ＜0.0001 | 0.0052 | 0.0002 | 0.7293 |

**P* < 0.05.

1. Adjusted for region, father’s ethnicity, father’s occupation, father’s education, child’s age, child’s sex, child’s BMI, childhood daily average outdoor physical activity durations and passive smoking.
