# Supplementary material for: Moderating effect of a sodium-rich diet on the association between long-term exposure to fine particulate matter and blood lipids in children and adolescents
Source: BMC Pediatr. 2024 Jul 20;24:466. doi: 10.1186/s12887-024-04896-8 (PMC11264876; doi:10.1186/s12887-024-04896-8)
Supplement: Supplementary file 2 — Supplementary Material 2. [file 12887_2024_4896_MOESM2_ESM.docx]

STROBE Statement—checklist of items that should be included in reports of observational studies

|  | Item No. | Recommendation | Page  No. | Relevant text from manuscript |
| --- | --- | --- | --- | --- |
| **Title and abstract** | 1 | (*a*) Indicate the study’s design with a commonly used term in the title or the abstract | 1-2 | Moderating effect of a sodium-rich diet on the association between long-term exposure to fine particulate matter and blood lipids in children and adolescents. A cross-sectional study. |
|  |  | (*b*) Provide in the abstract an informative and balanced summary of what was done and what was found | 2-3 | View the methods, results and conclusion sections of the abstract. |
| Introduction | | | |  |
| Background/rationale | 2 | Explain the scientific background and rationale for the investigation being reported | 2,4-6 | Several studies reported that exposure to higher levels of fine particulate matter (PM_2.5_) was associated with worse blood lipid levels in children and adolescents. However, whether a sodium-rich diet could modify the associations remains unknown. |
| Objectives | 3 | State specific objectives, including any prespecified hypotheses | 2 | To examine the associations of long-term exposure to PM_2.5_ with blood lipids in children and adolescents, and further examine the effect modification by dietary sodium consumption based on a multi-community population in China. |
| Methods | | | |  |
| Study design | 4 | Present key elements of study design early in the paper | 6 | a multi-community cross-sectional survey |
| Setting | 5 | Describe the setting, locations, and relevant dates, including periods of recruitment, exposure, follow-up, and data collection | 6-7 | A multi-community cross-sectional survey, which were conducted between November 2015 and December 2017, covering 5 urban districts and 9 rural counties in Sichuan Province, China. |
| Participants | 6 | (*a*) *Cohort study*—Give the eligibility criteria, and the sources and methods of selection of participants. Describe methods of follow-up  *Case-control study*—Give the eligibility criteria, and the sources and methods of case ascertainment and control selection. Give the rationale for the choice of cases and controls  *Cross-sectional study*—Give the eligibility criteria, and the sources and methods of selection of participants | 6-7 | We enrolled the participants aged from 6 to 17 years from a multi-community cross-sectional survey. In each region, two communities from urban districts or two villages from rural counties were selected using the systematic sampling (SS) method. In each district or village, one primary school and one middle school were selected by the simple random sampling (SRS) method. In addition, one senior high school was randomly selected from each district or county by the SRS method. Finally, one class was randomly selected from each grade, and 28 students were SS selected from each class to participate in the study. |
|  |  | (*b*) *Cohort study*—For matched studies, give matching criteria and number of exposed and unexposed  *Case-control study*—For matched studies, give matching criteria and the number of controls per case |  | NA |
| Variables | 7 | Clearly define all outcomes, exposures, predictors, potential confounders, and effect modifiers. Give diagnostic criteria, if applicable |  | NA |
| Data sources/ measurement | 8* | For each variable of interest, give sources of data and details of methods of assessment (measurement). Describe comparability of assessment methods if there is more than one group | 7-10 | Blood lipid biomarkers included blood TC, HDL-C, LDL-C, and TG. PM_2.5_ exposures were measured by the Information of participants’ address for schools. A standardized semi-quantitative Food Frequency Questionnaire (FFQ) was used to measure dietary sodium levels. Random urine samples were used to examine the urinary sodium. |
| Bias | 9 | Describe any efforts to address potential sources of bias | 8,10 | Blood lipid biomarkers and urinary sodium levels were examined by experienced clinical laboratorians according to the standard operating procedures of the instruments in the laboratory of the study hospitals. |
| Study size | 10 | Explain how the study size was arrived at | 6-7 | We enrolled the participants aged from 6 to 17 years from a multi-community cross-sectional survey. |

Continued on next page

| Quantitative variables | 11 | Explain how quantitative variables were handled in the analyses. If applicable, describe which groupings were chosen and why | 10 | We summed up the distribution of demographic, social-economic, and behavioral characteristics of the study participants across urinary sodium quartiles by count and proportion. |
| --- | --- | --- | --- | --- |
| Statistical methods | 12 | (*a*) Describe all statistical methods, including those used to control for confounding | 11 | Linear regression models were applied to explore the correlations of exposure to PM_2.5_ with blood lipid outcomes. We conducted the stratified analysis by dietary sodium quartiles and urinary sodium quartiles. In each stratum, a linear regression model was fitted in order to explore the associations of PM_2.5_ with blood lipid outcomes. |
|  |  | (*b*) Describe any methods used to examine subgroups and interactions | 11 | We conducted the stratified analysis by dietary sodium quartiles and urinary sodium quartiles. In each stratum, we fitted a linear regression model to analyze the associations of PM_2.5_ with blood lipid outcomes. To obtain an overall assessment of effect modification, we applied the likelihood ratio test, comparing the difference between the model with the interaction term and the model without that interaction term. |
|  |  | (*c*) Explain how missing data were addressed | 7 | The participants with missing data were excluded. |
|  |  | (*d*) *Cohort study*—If applicable, explain how loss to follow-up was addressed  *Case-control study*—If applicable, explain how matching of cases and controls was addressed  *Cross-sectional study*—If applicable, describe analytical methods taking account of sampling strategy |  | NA |
|  |  | (*e*) Describe any sensitivity analyses | 11 | We conducted several sensitivity analyses to assess robustness of the study associations. We first evaluated the dietary variations on the study associations by additionally adjusting for the dietary intake of total fat and energy. Finally, we excluded the participants whose parents have been diagnosed with cardiovascular diseases to evaluate the genetic variations on the study associations. |
| Results | | | | |
| Participants | 13* | (a) Report numbers of individuals at each stage of study—eg numbers potentially eligible, examined for eligibility, confirmed eligible, included in the study, completing follow-up, and analysed | 7 | 3711 participants were included in the main analyses. The stratified analysis using dietary sodium data and urinary sodium data included 3635 and 3631 participants respectively. |
|  |  | (b) Give reasons for non-participation at each stage | 7 | The participants with missing blood lipids results (n=73) were excluded. In the stratified analyses, participants missing dietary sodium information (n=76), or urinary sodium information (n=80) were also respectively excluded. |
|  |  | (c) Consider use of a flow diagram | 7 | Supplementary Material Figure S1 |
| Descriptive data | 14* | (a) Give characteristics of study participants (eg demographic, clinical, social) and information on exposures and potential confounders | 11 | Table 1 |
|  |  | (b) Indicate number of participants with missing data for each variable of interest | 7 | blood lipids results (n=73) , dietary sodium information (n=76), or urinary sodium information (n=80). |
|  |  | (c) *Cohort study*—Summarise follow-up time (eg, average and total amount) |  | NA |
| Outcome data | 15* | *Cohort study*—Report numbers of outcome events or summary measures over time |  | NA |
|  |  | *Case-control study—*Report numbers in each exposure category, or summary measures of exposure |  | NA |
|  |  | *Cross-sectional study—*Report numbers of outcome events or summary measures | 7 | 3711 participants were included in the main analyses. |
| Main results | 16 | (*a*) Give unadjusted estimates and, if applicable, confounder-adjusted estimates and their precision (eg, 95% confidence interval). Make clear which confounders were adjusted for and why they were included | 12 | Table 3 |
|  |  | (*b*) Report category boundaries when continuous variables were categorized | 12 | The distribution of long-term exposure to PM_2.5_ ranged from 22.4 to 65.0 μg/m^3^ among the study participants (Table 2). |
|  |  | (*c*) If relevant, consider translating estimates of relative risk into absolute risk for a meaningful time period |  | NA |

Continued on next page

| Other analyses | 17 | Report other analyses done—eg analyses of subgroups and interactions, and sensitivity analyses | 14 | Figure 2, Supplementary Material Table S3, Supplementary Material Table S4 |
| --- | --- | --- | --- | --- |
| Discussion | | | | |
| Key results | 18 | Summarise key results with reference to study objectives | 14 | The results showed that exposure to higher levels of ambient PM_2.5_ was associated with higher blood TG, LDL-C, and lower HDL-C levels. Most importantly, we provided a consistent evidence linking a high-sodium diet modified the associations of PM_2.5_ exposure with blood lipids in children and adolescents. |
| Limitations | 19 | Discuss limitations of the study, taking into account sources of potential bias or imprecision. Discuss both direction and magnitude of any potential bias | 17 | First, the nature of cross-sectional study prevented us from establishing causal relationships for the study associations. Second, limited by the data availability, we only evaluated the long-term PM_2.5_ exposure based on schools, which missed the residential exposures. A further study should replicate our analyses with allowing for the exposures from residence. |
| Interpretation | 20 | Give a cautious overall interpretation of results considering objectives, limitations, multiplicity of analyses, results from similar studies, and other relevant evidence | 15-16 | In this study, based on dietary estimates and urinary biomarkers, we provided consistent evidence that a lower sodium diet could reduce the toxic effects of PM_2.5_ on blood lipids in children and adolescents. |
| Generalisability | 21 | Discuss the generalisability (external validity) of the study results | 16 | Since the important role of abnormal lipid levels in predicting the subsequent risks of incident ASCVDs, our findings provide a new insight to treat the low-sodium diet as a preventive method to reduce the risk of incident ASCVDs from PM_2.5_ exposure in children and adolescents. |
| Other information | |  | | |
| Funding | 22 | Give the source of funding and the role of the funders for the present study and, if applicable, for the original study on which the present article is based | 20 | This work was supported by the Talent Introduction Scientific Research Projects Funded Start-Up Funds of Sichuan University [YJ2021120]. |

*Give information separately for cases and controls in case-control studies and, if applicable, for exposed and unexposed groups in cohort and cross-sectional studies.

**Note:** An Explanation and Elaboration article discusses each checklist item and gives methodological background and published examples of transparent reporting. The STROBE checklist is best used in conjunction with this article (freely available on the Web sites of PLoS Medicine at http://www.plosmedicine.org/, Annals of Internal Medicine at http://www.annals.org/, and Epidemiology at http://www.epidem.com/). Information on the STROBE Initiative is available at www.strobe-statement.org.
